# Dr. Devi Prasad Shetty: The Visionary Cardiothoracic Surgeon Who Championed Affordable Healthcare

**DOI:** 10.7759/cureus.70683

**Published:** 2024-10-02

**Authors:** Dheeraj Jayakumar, Anushree Bansal, Joselv E Albano, Janelle Lara G Mirhan

**Affiliations:** 1 Surgery, Davao Medical School Foundation, Inc., Davao City, PHL; 2 Neurosurgery, Southern Philippines Medical Center, Davao City, PHL; 3 Obstetrics and Gynecology, Brokenshire Medical Center, Davao City, PHL

**Keywords:** affordable healthcare, biography, cardiothoracic surgery, devi prasad shetty, historical vignette, narayana, yeshasvini

## Abstract

Dr. Devi Prasad Shetty, an Indian cardiothoracic surgeon, is known for his efforts in making sure that quality healthcare can be affordable and accessible for every single individual, not only in India but all over the globe. He graduated in medicine from Mangalore and continued his surgical training in London. After returning to India, he performed the first neonatal heart surgery in the country and was the personal physician for Mother Teresa. He helped start the Yeshasvini Micro-Health Insurance Scheme and formed Narayana Health, revolutionizing overall accessibility to healthcare. Through the development of digital health platforms and pioneering surgical techniques, he transformed patient care in India with his innovative ideas. His legacy lives on, motivating the next generation to work for the mission of affordable healthcare.

## Introduction and background

This review aims to highlight the significant contributions and efforts made by Dr. Devi Shetty (1953-present), an Indian cardiothoracic surgeon, in providing accessible healthcare to the masses in India and increasingly across the globe. He is the known founder and leader of Narayana Hrudayalaya, a tertiary privately owned hospital system, which over the years has evolved into Narayana Health, a massive network of 47 private healthcare facilities strategically located across India.

This has brought quality medical care closer to the underprivileged communities of the nation [1]. His life has been deeply influenced by Mother Teresa, who had a profound impact on him and served as an inspiring force behind the establishment of Narayana Hrudayalaya. His visionary leadership to make quality healthcare affordable for all has drawn global attention. His extraordinary progression from a distinguished cardiac surgeon to a prominent entrepreneur exemplifies his steadfast commitment and visionary leadership, especially in utilizing economies of scale to lower healthcare delivery costs in India [2].

## Review

Early life and education

**Figure 1:**
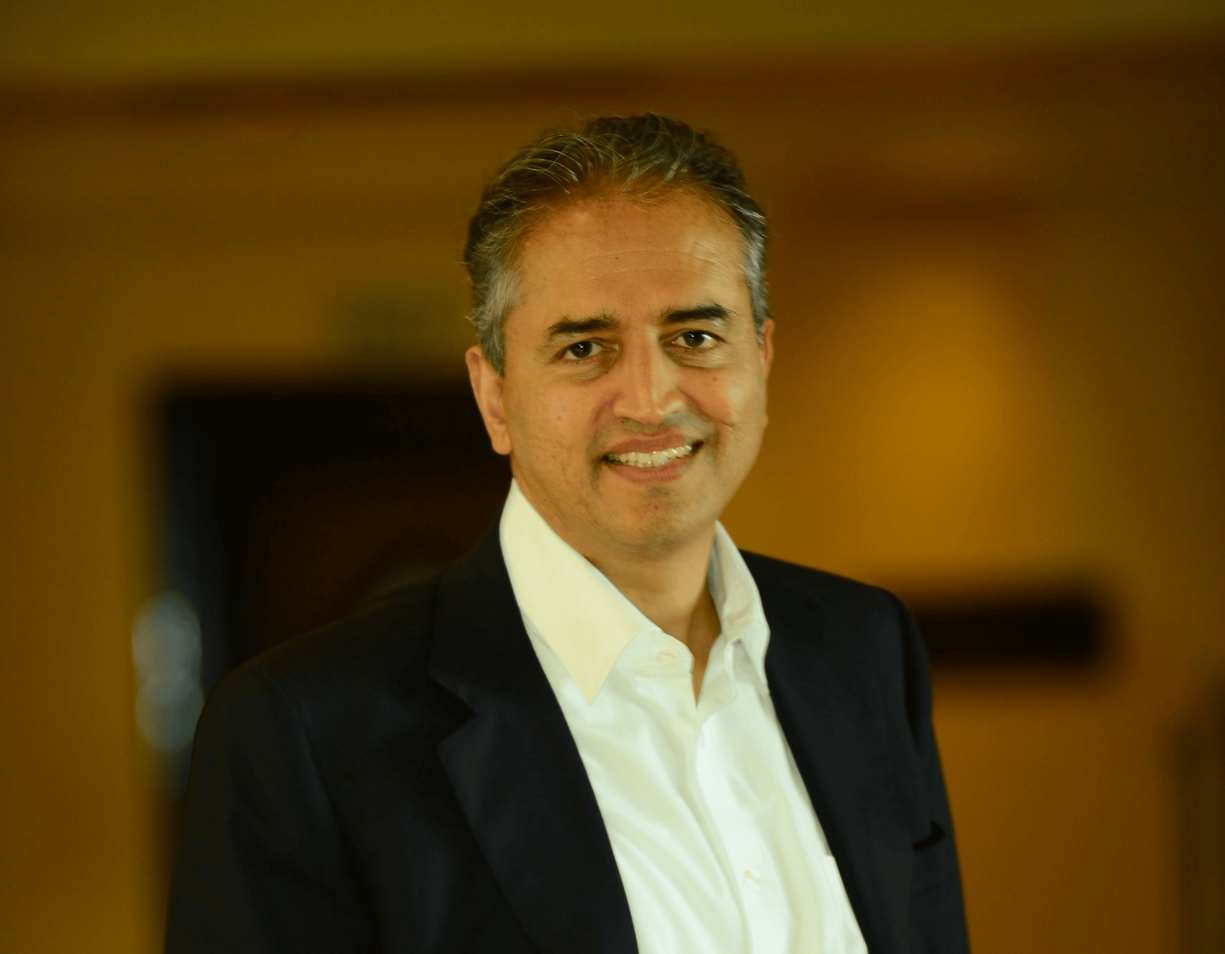
Dr. Devi Prasad Shetty Image credit: Indian School of Business [1]; Fair Use

Dr. Devi Prasad Shetty was born on May 8, 1953, in a small village of Kinnigoli and raised in the state of Karnataka, India (Figure 1) [2]. He was the eighth of nine siblings. His interest in cardiac surgery was sparked during his primary school years. In an interview done with Harvard Business School, Dr. Shetty fondly recalls his inspiration for this field as the day his primary school teacher came to his class and announced that Dr. Christiaan Barnard had his first heart transplant [3].

Academic pursuits

Dr. Shetty received his education at St. Aloysius School in Mangalore and pursued his Bachelor of Medicine and Bachelor of Surgery from Kasturba Medical College, Mangalore, in 1979. Later, he went on to obtain his postgraduate studies in general surgery at the same institution in 1982. Following his dream of becoming a cardiac surgeon, Dr. Shetty trained at the Guy's Hospital in London, United Kingdom, for cardiothoracic surgery [3].

Upon returning to India in 1989, he commenced his work at B.M. Birla Hospital in Kolkata. In 1992, he accomplished a significant milestone by performing India's first neonatal heart surgery on a 21-day-old infant [4]. Additionally, Dr. Shetty served as the personal physician to Mother Teresa in the year 1996, performing angioplasty on her following a myocardial infarction [5]. He is a fellow of the Royal College of Surgeons in the United Kingdom and received honorary doctorates from the University of Minnesota Medical School, USA, and Indian Institute of Technology Madras, India [2,6].

Leadership and recognition

Dr. Shetty led the Yeshasvini Micro-Health Insurance Scheme in Karnataka to make healthcare accessible and affordable for the underprivileged. Launched in 2003, the scheme was designed to help farmers and peasants access medical care at a nominal premium of 5-7 Indian Rupees (approximately 6-8 cents in US dollars) per month [4]. Yeshasvini covers a wide range of medical treatments, including major surgeries, thus significantly reducing the financial burden on the economically disadvantaged. The success of Yeshasvini has inspired similar schemes in other Indian states, such as the Rajiv Arogyasree in Andhra Pradesh and the Kalaingar Insurance in Tamil Nadu, conclusively showing the impact of proper leadership and delegation of an equitable scheme [7]. His expertise in his field has led to the invitation by the National Board of Examinations as an independent examiner for fellowship students in cardiac surgery. This role involves evaluating the competencies and skills of upcoming cardiac surgeons in India, ensuring they meet the high standards required for practice in this critical field [8].

He has received numerous prestigious awards for his contributions to medicine and public welfare. In 2012, he was honored with the Padma Bhushan, India's third-highest civilian award, while earlier in 2004, he received the Padma Shri [9]. In 2003, Dr. Shetty was recognized with multiple honors, including the Ernst & Young Entrepreneur of the Year award in the start-up category [2]. His innovative approach to reducing healthcare costs using mass-production techniques by allowing medical staff trained in India and other overseas countries to practice in the Cayman Islands earned him the Economist Innovation Award in Business Process in 2011 and the Entrepreneur of the Year award at the Economic Times Awards in 2012 [10].

The Narayana Medical Center

In 2000, Dr. Shetty initiated a groundbreaking venture by establishing Narayana Hrudayalaya, a comprehensive hospital located in Bommasandra on the outskirts of Bangalore. With a strong commitment to economies of scale, he aspired to cut healthcare costs by 50% within the next 5-10 years. His venture features a variety of specialized departments, such as cardiology, neurosurgery, pediatric surgery, hematology, transplant services, nephrology, and others. Remarkably, the heart hospital is one of the largest in the world, with 1,000 beds, performing more than 30 major heart surgeries daily [3]. The hospital, constructed on reclaimed marshland, has evolved into a major health city, accommodating approximately 15,000 outpatients daily. In his own words, he envisioned a hospital where "one need not be rich to get treated, and one shouldn't be poor and suffer" [3]. Over the span of two decades, Narayana Hrudayalaya evolved into Narayana Health, with over 47 healthcare centers located across India. Narayana Health has made quality medical services and care accessible to underprivileged communities.

Furthermore, the inclusion of hospitals in the Cayman Islands showcases the organization's global reach [2]. Dr. Shetty considers the Narayana Medical Center a stepping stone to holistic patient healthcare. In an interview published by the American College of Cardiology, he is quoted to have said that, "It is pointless for a hospital to be boasting about its academic and clinical excellence without looking at its patients holistically, and this includes an awareness about their backgrounds and their ability to pay" [11].

Patient healthcare at a fraction of the cost

The Yeshasvini scheme introduced in Karnataka, India, was mainly focused on healthcare accessibility. Research conducted on this scheme revealed that ensuring health security for large segments of the population in developing countries relies more on effective mobilization and organization than on resources alone [12]. While healthcare infrastructure is necessary, it is not sufficient on its own, and a sufficiently large subscriber base would be needed to develop infrastructure [12]. This scheme then paved the way for more accessible healthcare costs.

A long-term initiative of Dr. Shetty was to make healthcare accessible to everyone, and he devised an algorithm in 2018 and patented it as a "System and Method for Facilitating Delivery of Patient Care" [13]. In this, he employed the use of digital health platforms, patient monitoring systems, electronic health records, telemedicine tools, and decision support systems. These methods were aimed at improving care coordination, communication between providers and patients, remote monitoring capabilities, data analytics for personalized treatment plans, and overall efficiency in delivering quality patient-centered care [9]. These systems are vital in optimizing clinical workflows while prioritizing patient safety and well-being throughout their healthcare journey. His innovative approach to healthcare delivery has been a game-changer in the industry [14].

Novel techniques in cardiothoracic surgery

Dr. Shetty has made significant contributions to the field of cardiothoracic surgery. He developed a novel treatment for pulmonary hemorrhage during thromboendarterectomy surgery in which he performed selective segmental pulmonary artery ligation and reconstruction [15]. Although this is a rare condition, Dr. Shetty developed a method that involved irrigation of the pulmonary artery, creation of a hemostatic plug, and application of the hemostatic agent in a unique way that prevents further bleeding. Comparative analysis with prior techniques indicated that liters of blood were collected during surgery, but this novel technique has resulted in only 5-10 mL of blood loss at a time [15].

In addition to this, he also innovated a technique to address post-septal myectomy ventricular septal defects (VSD) wherein he developed a rapid autologous pericardial patch repair of the VSD created during septal myectomy for hypertrophic obstructive cardiomyopathy [16]. VSD following septal myomectomy is rare; it typically enlarges after surgery and is often detected through postoperative transthoracic echocardiography [17,18]. Dr. Shetty developed this technique that utilized a biventricular approach, which provides excellent exposure and enables precise closure of the defect with a custom-made, dumb-bell-shaped polytetrafluoroethylene (PTFE) device, thereby minimizing the risk of a residual VSD. This allowed for reduced mortality and complication rates previously involved with septal myomectomy [16].

## Conclusions

Dr. Devi Shetty's journey exemplifies his relentless efforts to make healthcare both accessible and affordable. Motivated by the example of Mother Teresa, he has persistently worked to lessen the gap between quality healthcare and affordability, making a profound impact on countless lives in India and beyond. Narayana Health, a manifestation of his vision and dedication, stands today as a beacon of comprehensive healthcare services, significantly enhancing the standard of medical care in India. Dr. Shetty's journey is an illustration of the transformative impact one individual can have on society, and his enduring legacy continues to inspire future generations of physicians.
